# Revealing the Missing Person Within the Data: Bottom-Up Development of Patient-Centred, Integrated Care Pathways from Fragmented Health Records: A Retrospective Case Study

**DOI:** 10.5334/ijic.10175

**Published:** 2026-11-03

**Authors:** Sonja Cassidy, Ove Lintvedt, Francis Odeh, Conceição Granja, Terje Solvoll

**Affiliations:** 1 Department of Strategic ICT, Helse Vest IKT, Bergen, Norway; 2 Faculty of Nursing and Health Sciences, Nord University, Bodø, Norway; 3 Norwegian Centre for E-health Research, University Hospital of North Norway, Tromsø, Norway; 4 Department of Health and Social Care, Municipality of Bodø, Bodø, Norway; 5 Department of Community Medicine, UiT –The Arctic University of Norway, Tromsø, Norway; 6 Norwegian Centre for E-health Research, University Hospital of North Norway, Tromsø, Norway; 7 Norwegian Centre for E-health Research, University Hospital of North Norway, Tromsø, Norway

**Keywords:** patient-centred care, integrated care pathways, patient perspective, healthcare services design, mental health, electronic health records

## Abstract

**Background:**

Access to patient-provided perspectives in Electronic Health Records (EHR) is essential for supporting patient-centred, integrated care, yet such information remains underrepresented. This study explores how patient perspectives are represented in health records and how their integration could support integrated care pathways.

**Research methods:**

This retrospective case study utilised hermeneutics, pragmatism, and sociotechnical systems theory to analyse 1,116 healthcare contacts from the records of 14 patients with co-occurring mental and physical health conditions. Deductive–inductive thematic content analysis was applied to identify and present the documented patient perspectives systematically.

**Results:**

Patient-centred information, such as personal goals and life events, was inconsistently recorded in unstructured formats, complicating the coordination of care across institutional silos.

**Discussion:**

The fragmentation of patient perspectives reflects a disconnect between national policy intent and practical implementation. Implementing a virtual health record model alongside a standardised ontology could unify relevant information from disparate healthcare settings, ensuring data remains meaningful and consistent.

**Conclusion:**

Integrating patient perspectives into EHRs is vital for informed decision-making and continuity of care. Overcoming persistent systemic, organisational, and technical barriers requires a coordinated approach involving policy reform and technological standardisation.

## 1. Introduction

The global increase in life expectancy has led to a growing number of individuals living with chronic conditions, putting additional strain on healthcare systems [1]. Vulnerable populations, such as adults with mental health conditions, are at higher risk of physical diseases and often receive fragmented care, increasing the risk of disability and early death [2,3]. Addressing these needs requires a shift from disease-focused care to models that integrate direct reports of health without provider interpretation; the *patient perspective* [4].

Building on the foundation proposed in our previous framework for Patient-centred, Integrated Care Pathways (PCICPs), which emphasises placing the patient at the centre of care, this approach advocates for including patient perspectives as essential data [5]. At the national or system level (macro) and organisational (meso) levels, it allows for more effective planning of patient-centred services and helps develop care pathways that support continuity of care across systems [6,7]. At the individual level (micro), patient-centred care recognises each patient’s unique needs, goals and experiences as expert knowledge, allowing for more informed and personalised care decisions [8,9].

*Care pathways* coordinate disease-specific interventions across care levels, differing from *clinical guidelines* that translate evidence into institutional practice [10]. In contrast, *patient pathways* reflect individual experiences in navigating the healthcare system [11]. Patients with complex health needs interact with multiple settings, leaving an *information trail* across various EHR systems [12]. However, current Health Information Systems (HISs), including EHRs, focus on disease-specific interventions and lack the capacity to include patient-reported data, such as social determinants or subjective experiences [13,14]. This omission leaves the patient perspective, the *missing person*, underrepresented in care decisions [15].

Registering patient-centred information is essential for integrated care, yet current EHRs often fail to capture these narrative views, and no consensus exists on incorporating this perspective. Building on our previously proposed framework [5], this study examines the challenges associated with integrating patient-provided data within fragmented EHRs at micro, meso, and macro levels. Patients experiencing co-occurring mental and physical health issues were selected as the focus group due to their complex needs, enabling a thorough evaluation of the framework’s adaptability. We outline patient perspectives for inclusion in care pathways, assess their representation across EHR systems, and illustrate information flows using PCICP models [5]. Finally, we propose solutions to bridge these gaps, advancing our framework’s operationalisation. In doing so, this study seeks to reveal the *missing person* in EHR systems and propose strategies to enhance patient-centred data integration.

### 1.1. In search of common ground

*Health* can be characterised as a fuzzy concept with multiple, sometimes conflicting, interpretations. The World Health Organization (WHO) defines it as being *a state of complete physical, mental and social well-being and not merely the absence of disease or infirmity* [16]. Ontologically, health might be viewed as the capacity to live fully and maintain well-being over time [17,18], understood in the context of how individuals feel, move, and engage in daily life [19]. Epistemologically, *health* may be conceptualised scientifically as a measurable, objective state systematically structured within EHRs. In contrast, a hermeneutic approach understands *health* through personal narratives and social contexts reflecting individual care journeys [19,20]. The integration of patient narratives into health information systems underscores a critical divide between clinical objectivity and a patient-centred understanding of health, thereby challenging the standardisation of patient narratives. Through a hermeneutic lens, both the patient and the various actors involved in their care from different domains and organisations bring their perspectives [19,21]. However, as argued by Dewey and Bentley, precision and clarity are essential for capturing these perspectives in a form that meaningfully supports care decisions [22]. From a socio-technical systems perspective, integrating patient perspectives into EHR systems requires attention to multiple levels of interaction (Figure 1). These include the capabilities and limitations of healthcare professionals and patients who interact with the EHR system, the organisational factors that shape workflows and patient pathways, and the broader regulatory environment in which the system operates as well as interactions between levels.

**Figure 1:**
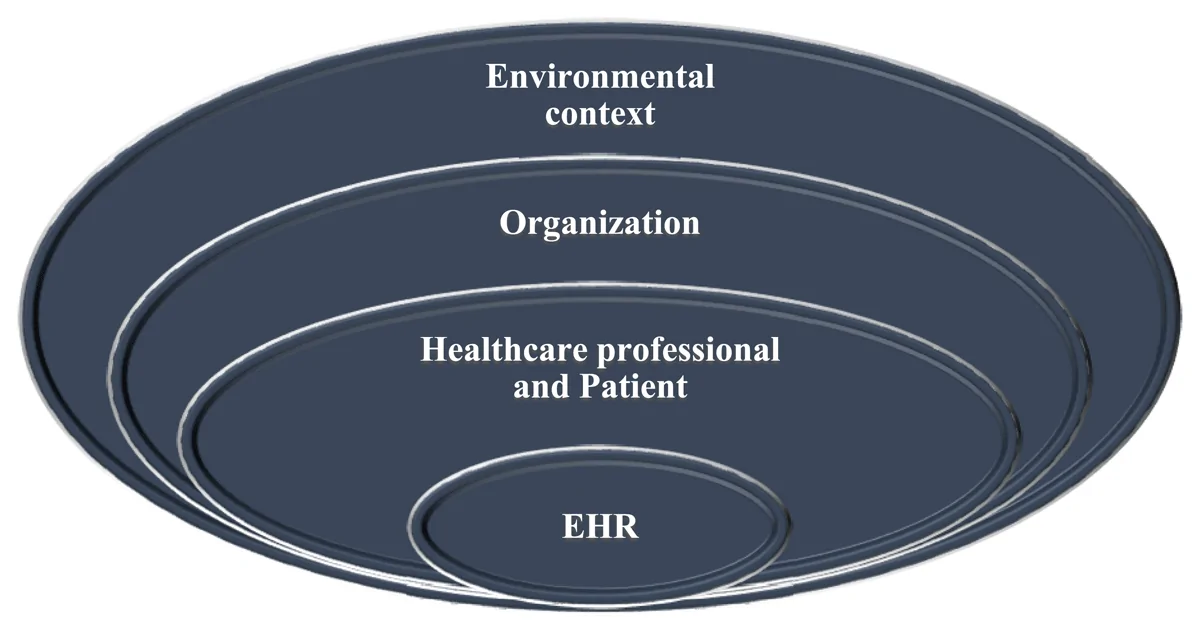
The levels of a complex socio-technical healthcare system. Adapted from Vicente, [23].

Integrating all perspectives into EHR systems poses challenges, especially in mental health contexts, where understanding relies heavily on the patient-provider relationship, care setting, and the patient’s willingness to share sensitive information [24].

### 1.2. A meeting of minds

Incorporating patients into shared decision-making is characterised by *voice*, *choice*, and *co-construction*, enhancing patient autonomy through active participation in care delivery. Integrating patient perspectives into care decisions requires re-evaluating traditional power dynamics, where the interplay of power, knowledge, and truth often dictates which perspectives are prioritised [25]. Ethnographic studies show that clinical decision-making is a shared, iterative process across settings, underscoring the importance of repeatedly including patient perspectives [14]. While advances in technology provide new opportunities to integrate these insights into workflows, participatory approaches remain underemployed in practice, particularly in mental health care [2,26].

### 1.3. The digital patient

Advancements in information technology facilitate a shift toward holistic, integrated care [27]. Digital tools such as patient portals enable the sharing of self-reported data, potentially enhancing care decision-making [28]. However, integrating patient-provided data into clinical workflows can be challenging due to time constraints and information overload. Standardising patient-reported data within EHR systems is complicated, and strict security measures can restrict data sharing across systems, risking the loss of patient perspectives at critical transition points [29,30]. Consequently, the potential for patient-provided information to inform care decision-making remains underutilised [13].

### 1.4. Getting lost on the path to integrated care

Implementing EHR-supported care pathways has been shown to enhance the quality of care and the utilisation of healthcare resources [31]. However, translating the strategic intent of incorporating patient perspectives into practical implementation remains challenging. Patient-centred care requires collecting and organising a richer array of patient information, regardless of when or by whom it was generated. This transition represents a socio-technical transformation and a shift in healthcare culture. Despite this, research on including patient perspectives to support integrated care pathways is limited [5]. Significant gaps remain in integrating patient experiences and preferences alongside clinical data for decision-making and service design. This constrains the development of pathways that accurately reflect the complex realities of individual journeys.

To address these gaps, this study employed a qualitative approach, bridging the theoretical understanding of patient perspectives with practical insights into how they are represented, or omitted, in EHRs. For integrated care practice, these findings provide an early indication of how embedding a patient’s longitudinal perspective into digital workflows can bridge cross-sector communication gaps. The following methods detail the steps taken to uncover the *missing person* within fragmented health records.

## 2. Research Methods

Methodologically, a hermeneutic approach was utilised to interpret human experience, meaning, and understanding within patient narratives [19,21]. Critical pragmatism framed the investigation as a value-driven inquiry, focusing on the operational utility of knowledge [22,32]. Finally, sociotechnical systems theory was applied to evaluate the system performance by addressing the inherent interdependencies between social workflows and technical EHR architectures [33].

We employed a retrospective case study to uncover *the missing person* in EHRs. Originally planned to combine archival analysis with patient feedback, this participatory approach became impossible due to COVID-19 restrictions. Consequently, the study became primarily qualitative, based on documented medical records, supported by a quantitative analysis of documentation patterns and healthcare interactions along the care pathway. This enabled us to trace patterns of inclusion and omission of patient-reported preferences, goals and experiences along care pathways, highlighting structural and contextual gaps in documentation practices.

### 2.1. Involvement of people with lived experience

This study investigated how patient experiences, goals, and priorities were captured in health records to highlight systemic documentation gaps. Due to strict COVID-19 access restrictions, direct participation from individuals with lived experience, including methodological decision-making, framework definition, and data interpretation, was not possible. We acknowledge this absence of direct participation as a methodological limitation, addressed further in Section 4.4. Despite this, our retrospective analysis of existing records allowed for a rigorous trace of how patient-reported preferences were documented across various healthcare levels.

### 2.2. Study context

The Norwegian healthcare system operates under a semi-decentralised model where state-funded authorities manage specialised care, and municipalities oversee primary and social services. Norway’s transition from paper files to electronic charting occurred within localised institutional boundaries rather than a unified framework. This has historically driven severe technical fragmentation and interoperability challenges [34]. To illustrate the scale of this divide, the Norwegian Directorate of Health reported 29 distinct EHR vendors operating 41 systems nationwide [35]. This distributed landscape contributes to the fragmented, unstructured nature of the historical medical records evaluated in this cohort, often forcing professionals to document a single patient journey across multiple, disconnected systems.

### 2.3. Aim and study design

The study consisted of three phases, as presented in Figure 2. Phase 1 investigated how patient perspectives were captured in historical medical records from general practitioners (GPs), urgent care facilities, home care services, and hospitals in northern Norway. Phase 2 identified gaps and deficiencies in EHRs concerning the patient perspectives on their care. Phase 3 visualised an exemplar case using the PCICP framework to demonstrate how patient views, priorities and health goals remained siloed across EHRs.

**Figure 2:**
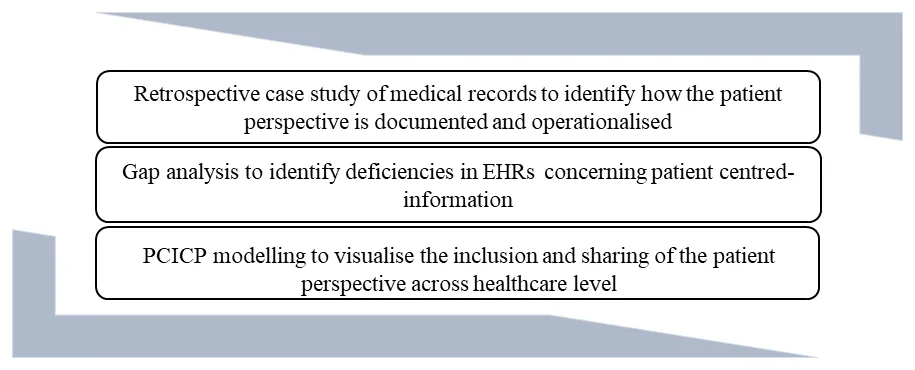
The 3-phased study design.

### 2.4. Study population

As access to healthcare personnel, patients, and current medical records remained restricted during the pandemic, the National Archives of Norway became an alternative source, providing data from deceased patients’ medical records predating 2007. A purposive sampling strategy was applied in the archive data of deceased Norwegian patients with documented mental and physical conditions, with a random selection of 70. Of those, the medical records of 20 patients were scanned, and 10 (five women and five men) were included for diversity in age, gender, and diagnoses. After the pandemic ended, convenience sampling of patients who had consented to provide access to their medical records resulted in the addition of the medical records of four living patients, bringing the total to eight females and six males. This mixed population enabled comparative analysis across healthcare systems and multiple EHR systems.

The records were collected from GPs, home care services, urgent care facilities and hospitals, including the Nordland Hospital Trust, the University Hospital of North Norway, and Bodø Municipality, alongside other agencies such as social services and law enforcement when relevant. All corresponding institutional ethical review board numbers, informed consent processes, and data de-identification protocols are detailed in Section 4.5.

### 2.5. Data extraction

The study examined retrospectively medical records from 14 patients with co-occurring mental and physical health conditions, a population typically requiring complex, cross-sector care. These records spanned the period from 1956 to 2024 and included documentation from multiple levels of care, institutions and disciplines, including general practitioners (GPs), specialist mental health and medical care in hospitals, community and home care services, and other agencies, such as social services and law enforcement. Data sources included notes and reports from both formal and informal care providers, referrals, appointment histories, summaries of patient-provider interactions across primary and specialised care, and records of contact with others involved in the patient’s care, such as family members, social services, or law enforcement.

Records were excluded if they were duplicated, lacked identifying information or contained only detailed clinical descriptions or medication lists without narrative content. We also excluded gender-specific conditions that could not be described in gender-neutral terms, rare conditions posing identification risks, isolated single-episode encounters, and administrative records regarding appointments or missing authorship.

To systematically identify and present the patient perspectives documented in the included records, an inductive-deductive thematic analysis was conducted guided by Braun and Clarke [36] phased approach. Despite the diversity of record media, ranging from scanned historical files to modern EHRs, all documents were treated uniformly, with relevant narrative text extracted into a standardised Excel worksheet to ensure identical exposure to the qualitative coding mechanism. Initial familiarisation involved iterative reading and extraction of relevant content to build a broad understanding of content and context. Relevant entries were categorised into micro, meso, and macro perspectives to ensure consistency in data extraction.

In the second phase, the initial coding of the patient narratives was theory-driven using three types of patient perspectives. *voice*, *choice*, and *co-production* [26]*. Voice* captured instances where patients expressed concerns, values, or reflections on their experience of care, and *Choice* entries where patients expressed their preferences or requests*. Co-production* referred to patient engagement in their care, such as shared decision-making or self-management activities. To ensure conceptual alignment and minimise bias, a multi-step verification process was used. The primary author and a clinically trained co-author independently coded a 10% preliminary sample to test the scheme’s stability. Discrepancies were resolved through team discussions until consensus was achieved, after which the primary author completed the dataset, supported by ongoing peer debriefing sessions with the co-authors to audit emerging categories.

In the third phase, codes were collated into overarching categories by identifying patterns in how patient concerns were reported, documented, and shared across settings. These categories were reviewed for internal consistency and alignment with original definitions, refined where necessary. To ensure continuous adherence to the established framework, the primary author conducted the primary review, selection, and mapping of content, while a clinically trained co-author periodically audited a subset of the finalised records using the consensus rules established during the preliminary sample phase. The steps are summarised in Table 1 below.

**Table 1:** Phase summary.


PHASE	DESCRIPTION	KEY FOCUS

**Familiarisation**	Iterative reading and extraction	Broad understanding of patient narratives and documentation context

**Initial coding**	Theory-driven coding (Voice, Choice, Co-production)	Identifying patient perspectives and engagement levels

**Category development**	Collating codes by identifying data patterns	Mapping concerns and documentation across settings

**Review and refinement**	Consistency and definition alignment	Ensuring coherence and validity of categories


Categorised narratives were mapped to the conceptual framework from the International Standardisation Organisation (ISO), standard *13940:2015, System of concepts to support continuity of care* [37], to support a structured representation of the patient’s perspective. This European standard offers a semantic model of care as relational, contextual, and evolving over time, and was selected to facilitate a way to structure the patient insights and aiding its inclusion into EHRs. Although the standard primarily focuses on clinical and administrative data, it was found to accommodate patient-centred information through concepts such as *Health matter*, *Health goal*, *Health thread*, *Non-ratified healthcare information*, and *Health need*, which correspond well with the patient narratives identified in this study.

The relationship between the three patient perspective categories, the types of narrative content extracted, and the corresponding ISO 13940 conceptual containers is presented in Table 2 below.

**Table 2:** Structured patient perspectives.


PATIENT PERSPECTIVE CATEGORY	NARRATIVE FROM THE MEDICAL RECORD	ISO 13940 CONCEPTUAL CONTAINER

**Voice**	Expressed values or wishes about service delivery	Health need

**Choice**	Patient’s treatment preference	Health goal

**Co-production**	Self-management actions	Healthcare activity


The final phases of analysis involved synthesising and reporting the categorised and mapped content using tables and descriptive summaries, including frequencies and percentages.

### 2.6. Data collection and content analysis

Data sources included notes and reports from both formal and informal care providers. This included referrals, appointment histories, and summaries of patient-provider interactions across primary and specialised care, as well as records of contact with others involved in the patient’s care, such as family members, social services, or law enforcement. Records that were duplicated, lacked identifying patient information, or contained only detailed clinical descriptions or medication lists were excluded from the analysis. Furthermore, records describing diseases specifically associated with gender were omitted if they could not be described in gender-neutral terms, and rare conditions that could render the patient identifiable were also excluded. Records documenting single contacts related to a specific incident of injury or disease that appeared only once in the patient’s health record were omitted, along with records concerning changes or cancellations of a single appointment and documents that did not identify the author or intended recipient.

## 3. Results

This section presents the findings from our study. The findings are organised according to the identified themes: the representation of the patient perspective, information registration and flow, care activity integration and service planning and provision.

### 3.1. Case study findings

The case study findings analyse patient records to uncover patterns in care documentation and provider activities across multiple healthcare levels. This analysis highlights the complexities of integrating patient perspectives into care documentation, focusing on the breadth of information, the diversity of service providers involved, and the challenges of maintaining continuity and coherence in patient records over time.

### 3.2. Study characteristics

We analysed medical records from 1956 to 2024 for 14 patients, encompassing more than 5,000 pages, resulting in 1,116 contacts with healthcare services across various levels of care, with 663 being primarily medically related. All patients had diagnoses of both mental health conditions (e.g., depression and anxiety) and physical health conditions (e.g., cardiac issues or diabetes), with 127 contacts describing substance abuse challenges. On average, each patient had contact with care providers 79 times, involving various providers such as law enforcement, social care, correctional facilities, detox clinics, private care clinics, home care (both digital and in person), outpatient community mental health services, urgent care, and hospitals (both private and public). Primary care providers accounted for 42%.

A total of 121 records contained non-clinical information provided by patients, relating to their physical, practical, and emotional care desires, significant life events, support persons, and perceived quality of life and health goals, such as managing daily activities and remaining independent at home. The documents were predominantly free text and varied significantly, encompassing referral or discharge notes, reports from home care nurses, and radiology reports. Few electronic documents contained fields for reporting lifestyle information or what patients considered important (such as hospital daytime nursing reports). Mental health issues were noted when pertinent to the patient’s physical care. In addition, lists of medications, prior healthcare contacts (both private and public), and diagnoses were frequently copied into various clinical documents to ensure that care providers had important information available at the point of care.

Several coding systems for diseases were employed, with more similarities observed between GPs and urgent care. No formal references to standardised care pathways or clearly defined care sequences were identified. Only one reference was found where the patient explicitly shared their experiences with care transitions across healthcare levels. A summary of the study characteristics is presented in Table 3.

**Table 3:** Patient health conditions and healthcare actors involved.


PATIENT	HEALTH RECORDS INCLUDED	MENTAL HEALTH CONDITIONS	HEALTHCARE ACTIVITY PERIOD (MENTAL HEALTH)	PHYSICAL HEALTH CONDITIONS	HEALTHCARE ACTIVITY PERIOD (PHYSICAL HEALTH)	HEALTH CARE ACTORS INVOLVED

**1**	32	Bipolar disorder, depression, anxiety disorder, suicide-related behaviour, substance abuse	1980/2001	Cardiovascular disease, cancer, infectious conditions	1993/2001	Social services, law enforcement, private detoxification clinic, public outpatient psychiatric clinic, hospital (mental and physical), urgent care, ambulance services, general practitioner

**2**	66	Bipolar disorder, depression, anxiety disorder, post-traumatic stress disorder, psychotic disorder, suicide-related behaviour, substance abuse	1967/1994	Cardiovascular disease, gastrointestinal disorder, genitourinary condition, neurological condition	1967/1993	Social services, law enforcement, private detoxification clinic, hospital (mental and physical), general practitioner, informal caregivers

**3**	35	Depression, personality disorder, psychotic disorder, suicide-related behaviour, neurodevelopmental disorder, substance abuse	1990/2007	Trauma	2003/2007	Social services, law enforcement, correctional facilities, private detoxification clinic, public outpatient psychiatric clinic, (mental and physical), urgent care, ambulance services, general practitioner, informal caregivers

**4**	51	Depression, personality disorder, suicide-related behaviour	1997/2000	Neurological disorder, movement disorder, chronic respiratory condition, trauma	1985/2002	Physical rehabilitation centre, police, home care services, public outpatient psychiatric clinic, hospitals, urgent care, general practitioner, informal caregivers

**5**	23	Substance abuse	1987	Cardiovascular disease, ocular, musculoskeletal, endocrine conditions, infectious disease	1961/1993	Physical rehabilitation centre, private detoxification clinic, public outpatient psychiatric clinic, hospital (mental and physical), urgent care, ambulance services, general practitioner

**6**	30	Depression, personality disorder, suicide-related behaviour, substance abuse	2001/2003	Gastrointestinal condition, rheumatic disorder	1976/2003	Social services, law enforcement, home care services, ambulance, hospital, urgent care, general practitioner, informal caregivers

**7**	21	Depression, suicide-related behaviour, substance abuse	1993/1996	Cardiovascular disease, genitourinary condition	1956/2001	Social services, private detoxification clinic, home care services, hospital, general practitioner, informal caregivers

**8**	51	Depression, suicide-related behaviour, substance abuse	1980/1999	Cardiovascular disease, gastrointestinal disorder	1988/1994	Social services, law enforcement, private detoxification clinic, home care services, ambulance, hospital, urgent care, ambulant team, general practitioner, informal caregivers

**9**	20	Depression, personality disorder, suicide-related behaviour, substance abuse	1996/1998	Musculoskeletal trauma, infectious condition	1991/1998	Social services, law enforcement, private detoxification clinic, ambulance, urgent care, hospital (mental and physical), general practitioner, informal caregivers

**10**	22	Depression, suicide-related behaviour, substance abuse	1987/2001	Eating disorder	1987	Law enforcement, urgent care, hospital (mental and physical), general practitioner, informal caregivers

**11**	141	Depression, neurodevelopmental disorder	2019/2024	Traumas (physical and sexual), eating disorder, gastrointestinal disorder, genitourinary conditions, musculoskeletal trauma, infectious condition, upper respiratory tract issues	2013/2024	Public outpatient psychiatric clinic, hospital (mental and physical), urgent care, home care services, general practitioner

**12**	228	Depression, anxiety disorder, suicide-related behaviour, substance abuse	2006/2024	Musculoskeletal trauma, cancer, speech and language deficit, neurological disorder, gastrointestinal disorder, genitourinary conditions, infectious conditions	1995/2024	Physical rehabilitation centre, social services, county governor, public outpatient psychiatric clinic, hospital (mental and physical), urgent care, ambulance services, home care services, general practitioner, informal caregivers

**13**	230	Depression, anxiety disorder, suicide-related behaviour, substance abuse	2011/2024	Trauma, cancer, liver diseases, endocrine disorder, neurological disorder, gastrointestinal disorders, infectious conditions	1997/2024	Physical rehabilitation centre, social services, hospital (mental and physical), urgent care, ambulance services, home care services, general practitioner, informal caregivers

**14**	166	Depression, suicide-related behaviour	2022/2024	Cardiovascular disease, neurological disorder, gastrointestinal disorder, ocular condition	2005/2024	Physical rehabilitation centre, social services, hospital (mental and physical), urgent care, ambulance service, care home, general practitioner, informal caregivers


Ultimately, these metrics confirm that recorded patient perspectives remain rare and unstructured.

### 3.3. Qualitative and semantic analysis

Analysis followed the PCICP framework, employing an iterative thematic content analysis refined into overarching themes like life events and transition challenges. Coding reliability was ensured through multi-researcher review. This qualitative method enabled a contextualised understanding of how patient perspectives were documented across different disciplines and care systems.

The analysis applied three patient-perspective categories: 1) Voice, expressing concerns, reflections on care quality, or information requests, 2) Choice: capturing preferences, such as the desire to manage care at home, and 3) Co-production: Referring to patients engaging in self-management or jointly agreeing on care plans [26]. These patient narratives were then mapped to the ISO 13940:2015 semantic framework to demonstrate how patient-centred information could be structured and standardised. For instance, patient aims, like living independently, were categorised as *Health goals*, and expressed wishes about service delivery were mapped to *Health needs*. Information provided by patients but not yet verified by clinicians was represented as *Non-ratified healthcare information*, allowing unstructured narratives to be mapped into standardised, interoperable concepts.

### 3.4. Descriptive quantitative analysis

The final phases of analysis involved synthesising and reporting the categorised and mapped content using tables and descriptive summaries, including frequencies and percentages. A brief descriptive quantitative analysis was conducted to contextualise the patient narratives and make visible the extent of fragmented or missing patient-reported information. This analysis examined five key metrics across patients, providers, and settings (summarised in Table 4), including the number of providers per patient and the documentation rate of patient-centred information per provider and per patient.

**Table 4:** Descriptive quantitative summary.


#	METRIC	DESCRIPTION	SUMMARY STATISTICS

**1**	Providers per patient	Distinct providers (actor × setting) per patient	Mean 15 (median 6)

**2**	Patient-centred information per provider	Entries among providers with ≥1 entry in “Other patient-provided information”	Mean 2.5 (median 1)

**3**	Records per patient	Total records per patient	Mean 79.7 (median 43)

**4**	Patient-centred information per patient	Entries among patients with ≥1 entry in “Other patient-provided information”	Mean 8.5 (median 4)

**5**	Patient-centred entries by healthcare level	Classified by point of care: “hospital” = specialist; all others = primary	Primary 59, specialist 60, not specified 2 (n = 121)


Counts are based on 1,099 records after excluding 17 entries with both provider fields missing. *Provider* was operationalised as the healthcare actor initializing care or the point of care where documentation occurred. *Patient-centred information* refers to entries recorded in *Other relevant, patient-provided information*. These metrics highlight the scarcity and fragmentation of patient-reported entries within the total volume of clinical records. However, the limited amount of patient-reported information in the records constrained the scope for more extensive quantitative analysis.

### 3.5. Conceptualising the digital pathway

Based on the concepts outlined in the PCICP framework and the analysis of medical records, we developed a conceptual pathway model (Figure 3) to translate our empirical data into a structured format. While the clinical timeline and information blocks are empirically derived from the case material, the overarching pathway mapping serves as a conceptual extrapolation to visualise these systemic information flows.

**Figure 3:**
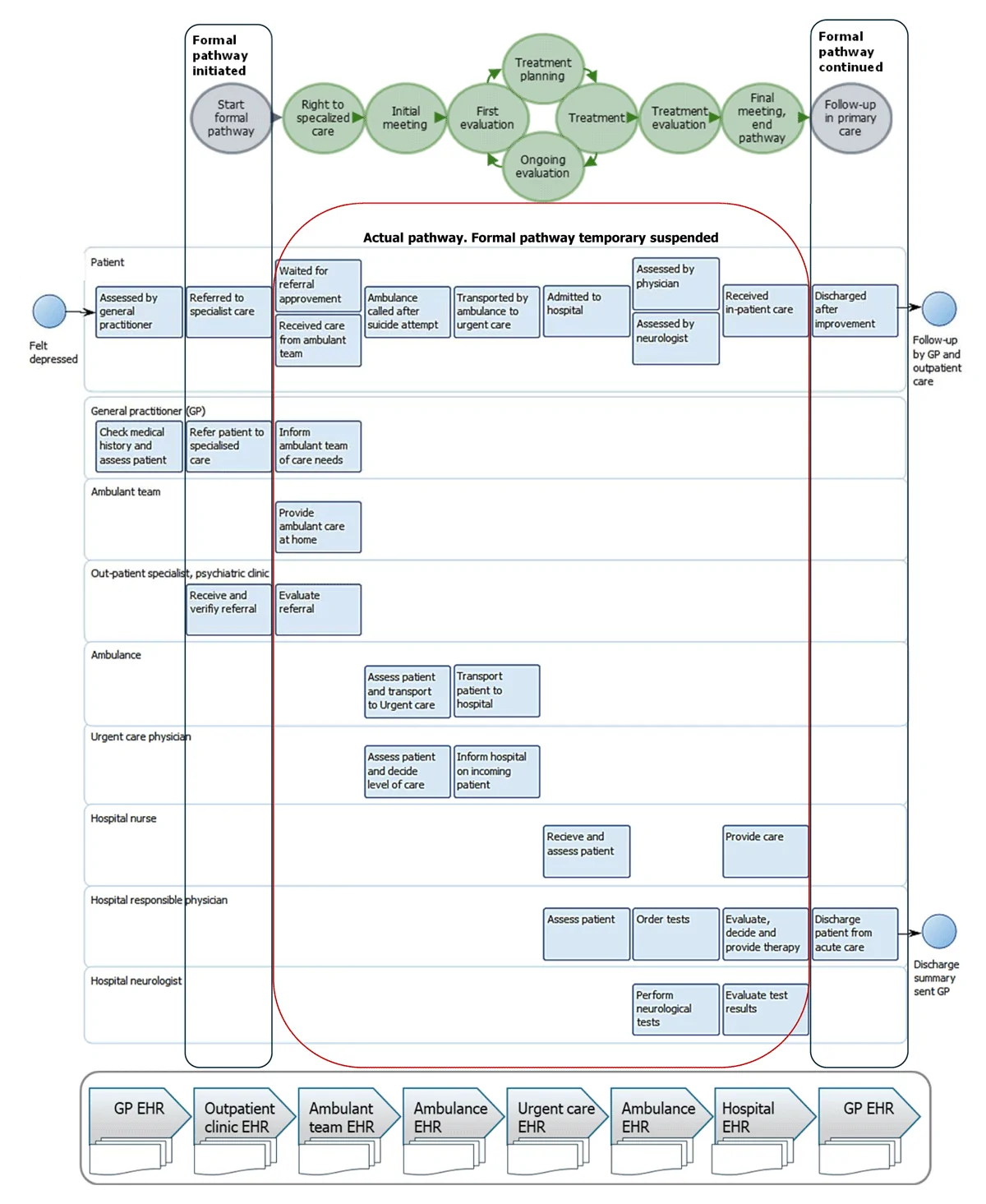
Conceptual Pathway Model including Fragmented EHR systems. Modelled using BPMN, Data Mapping Diagrams and QualiWare Lifecycle Manager 10.3.

The model represents, at the micro level, the patient’s actual care pathway across their various care providers, from the initial meeting with their GP, waiting to be referred to specialised care, then declining and requiring urgent care and hospitalisation. At the meso level, various healthcare levels and disciplines are involved in providing their care, from the GP and ambulant team providing the initial, outpatient care, to the ambulance, and the administrative and various healthcare professionals in the hospital, who were involved when the patient unfortunately needed to be admitted after attempting suicide. On the macro level, the standardised Norwegian National Pathway for Mental Health and Substance Abuse [38] includes a set of clinical activities for referring and treating patients suffering from depression.

However, while the formal pathway was initiated by the GP’s referral of the patient to specialised care (the activities “Start formal pathway” and “Deciding the right to specialised care”), the patient’s health declined, necessitating different care to be provided. Additionally, the model highlights the fragmented flow of information mirroring the clinical activities along the pathway. The various EHR systems used for collecting and documenting patient information are confined within institutional silos, resulting in the same information being recorded multiple times with different medical coding. Conclusively, this demonstrates how unintegrated EHRs segment a single journey, disrupting the continuity of the patient’s voice across the care pathway.

The results illuminate critical gaps in documenting and sharing patient perspectives across healthcare and IT systems. The discussion that follows contextualises these findings, highlighting implications for patient-centred, integrated care.

## 4. Discussion

The purpose of our study was to investigate the incorporation of patient perspectives within EHRs and identify gaps in capturing patient-centred data to support integrated care pathways across EHR systems. Our analysis of records from patients with mental and physical health conditions revealed significant gaps and inconsistencies in how these perspectives are recorded, impacting the continuity and comprehensiveness of patient information available to healthcare providers.

### 4.1. Implications of missing information at macro, meso, and micro healthcare levels

Our analysis revealed significant deficiencies in EHRs regarding patient-centred information, with implications at all levels of the healthcare system. At the macro level, our findings suggest a disconnect between the strategic intent of national policies and their practical implementation within organisations. Despite these policies, the underrepresentation of patient perspectives in EHR systems highlights the need for systemic changes to incentivise their inclusion. Furthermore, limited EHR interoperability and inadequate data-sharing regulations exacerbate these challenges, driving fragmentation and inefficiency.

At the meso level, gaps in organisational documentation practices hinder the standardisation and sharing of patient-centred data across systems, compromising continuity of care. When documented, patient-centred information is often unstructured, making it difficult for providers to access and use it effectively. Decisions regarding medication administration, food services, or rehabilitation training, whether prior, present, or planned, are crucial for all care providers to know, as they may influence the care administered or the medications provided to the patient. This is especially challenging for our focus group, as they frequently move between different levels of care. These issues reflect broader systemic challenges, such as inconsistent coding systems and a lack of standardised methods for documenting and sharing patient perspectives, hindering the establishment of a common understanding of when, how, or why patient perspectives should be included in care decisions.

At the micro level, patients’ needs for practical or emotional support, personal goals, and experiences were frequently missing or inconsistently recorded, excluding their perspectives from care decisions. Most data were retrospective and did not include planned activities, appointments, or care pathways, failing to provide a comprehensive view of the patient’s health and care journey. The study revealed that only one instance explicitly documented a patient’s experience with care transitions, highlighting the rarity of such crucial information in EHRs. Additionally, there was no comprehensive overview of who was currently involved in the patient’s care, risking misaligned care plans, reduced care quality, and poorer health outcomes. Critical information, such as a patient’s support network or personal circumstances, was known to one provider but not accessible to others, resulting in gaps in care continuity. This issue is especially pronounced for patients with both mental and physical health conditions, who often receive care from multiple providers and frequently transition between levels of care while needing to carry their health information across care levels and disciplines.

### 4.2. The international challenge

The challenges in this study are not unique to Norway; they reflect broader international patterns. Recent healthcare system evaluations by the OECD and WHO have highlighted a persistent gap between policy goals and practical implementation of person-centred, integrated care. Across many health systems, services remain fragmented, mechanisms for incorporating patient perspectives in care design and evaluation are limited, and digital infrastructures continue to prioritise clinical over experiential data [39,40,41]. These shortcomings are particularly evident for individuals with complex or long-term conditions and those with limited health or digital literacy [42], underscoring a global need for actionable models that can translate patient perspectives into meaningful contributions to care delivery.

### 4.3. Transforming fragmented health records into patient-centred, integrated care pathways

Building on our findings, this section proposes normative design recommendations to transform fragmented records into integrated pathways. The inconsistent documentation of patient perspectives in EHR systems continues to limit the ability to create comprehensive, patient-centred health records. As illustrated in Figure 3, patient information is often scattered across multiple systems, making it difficult to support the objectives of patient-centred, integrated care pathways. Achieving these objectives requires solutions that enable true interoperability and the secure, seamless sharing of patient data across the care continuum.

To address these challenges, the PCICP model was expanded by drawing on the work of Solvoll et al. (2023) and the Valkyrie research project [12,43]. The resulting Virtual Health Record (VHR) and updated PCICP model (Figure 4) is presented here not as an empirical finding of this study, but as a conceptual extrapolation designed to address the documentation gaps identified in our analysis. This architecture offers a practical approach to consolidating disparate data sources into a single, patient-centred view.

**Figure 4:**
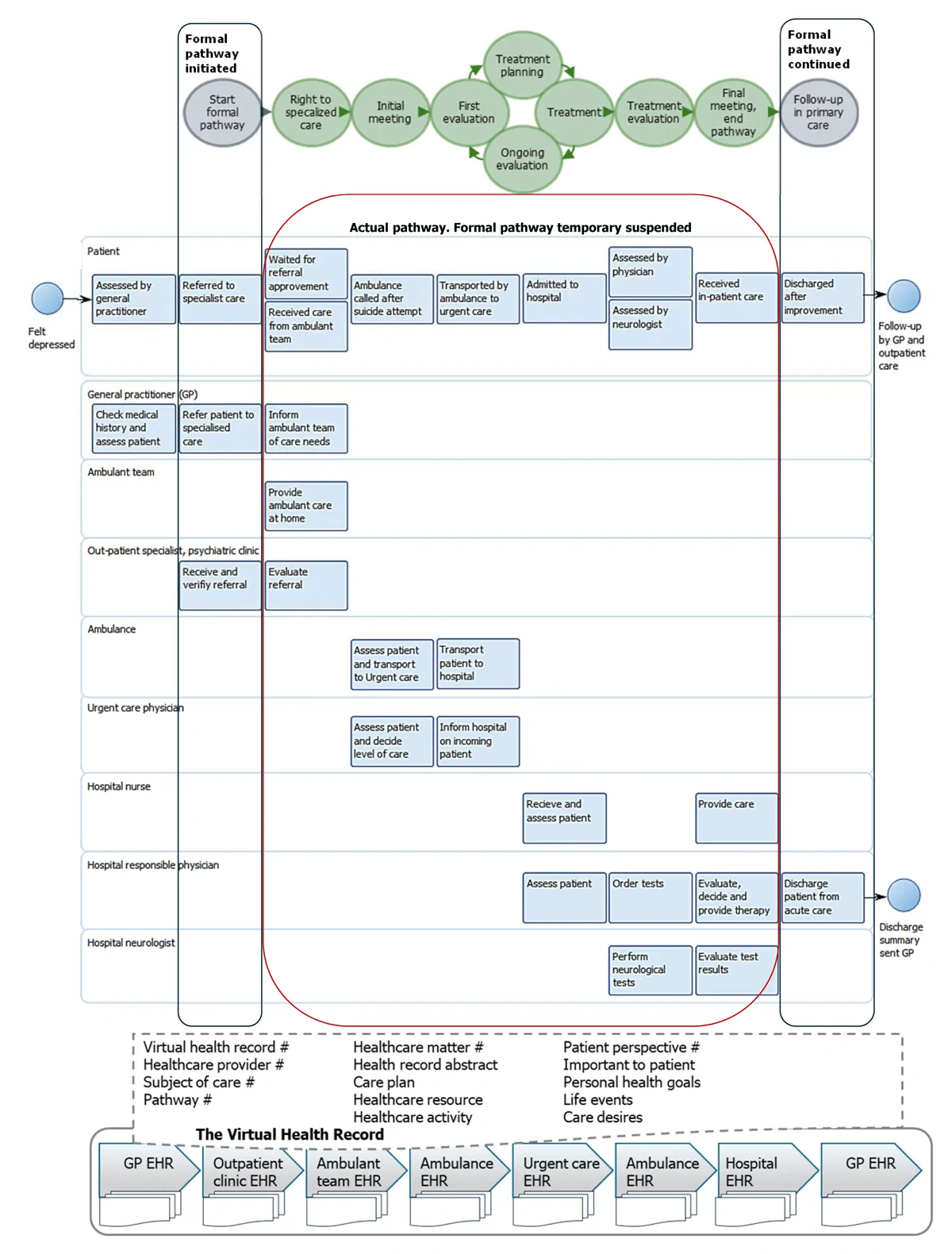
Conceptual pathway model supported by an integrated virtual health record. Modelled using BPMN and QualiWare Lifecycle Manager 10.3.

The PCICP model also incorporated relevant concepts from the ISO 13940:2015 standard, adapting elements such as *Health Thread* and *Health Goal* to represent patient perspectives in a structured and interoperable way, potentially reducing reliance on free text and enabling retrieval and reuse across care settings.

As shown in Figure 4, the VHR acts as a secure, coordinated view that brings together relevant information from multiple healthcare settings when needed. A care provider with a legitimate care relationship with the patient, such as the ambulant team in Figure 3, requires a more complete picture of the patient’s health journey. Using patient identification details, the VHR locates and links to records across the various EHR systems. Each system contributes patient-centred entries such as consultations, diagnoses, historic and planned treatments, and patient-reported information, which are then assembled into a unified, virtual record. To guarantee the accurate and relevant presentation of information for each patient and healthcare professional, standardised ontologies are employed, ensuring that data remains meaningful and consistent across the various EHR systems.

Figure 4 illustrates how integrating patient perspectives into the VHR model can transform fragmented records into a unified, meaningful resource. Compared to current, fragmented health records (Figure 3), the proposed model ensures that patient-reported experiences, needs, preferences, and goals —elements often missing or inconsistently documented in traditional EHRs —are directly linked to clinical events within the pathway, shared seamlessly across organisational and technological boundaries. Embedding these perspectives alongside structured clinical information enables care plans to be tailored to what is truly meaningful for the individual. It supports more informed decision-making, fostering continuity of care and facilitating a truly patient-centred approach to healthcare delivery.

### 4.4. Limitations

The findings of this study should be interpreted with certain limitations. Data were drawn from both scanned, free-text, paper-based records and more structured electronic health records, with evolving documentation practices, coding systems, and record formats [37]. The use of both older records from deceased patients and newer EHRs may have introduced inconsistencies due to evolving healthcare practices, coding systems, and documentation standards. Since healthcare professionals wrote most entries, there is also a risk that the documented patient voice does not fully reflect what the patients said or meant. In addition, patients have only more recently been permitted to review and amend their medical records [44]. Additionally, there is a risk of bias both at the *healthcare system level*, where patient selection and imperfect data capture may skew representation, and at the *research level*, where the design, extraction, analysis, and interpretation of data may influence findings [45]. Furthermore, structural changes and policy reforms in the Norwegian healthcare system have reshaped the way care is provided, documented and evaluated over the relevant period [46,47].

Health or digital literacy was not fully considered in the provision of patient-centred data. The small and heterogeneous sample focused on participants with complex, co-occurring mental and physical health conditions, which limits the transferability of the findings to wider populations but allowed for a deep-dive evaluation of longitudinal care journeys. These selection parameters inherently shaped our findings. While this effectively exposed cross-sectoral silos, it may shape our interpretation by overemphasise systemic fragmentation.

Ethical constraints, patient record sensitivity, and COVID-19 restrictions precluded validation through interviews, observations, or surveys of living patients. However, our focus on existing EHR data provided a bio-socio-technical perspective, integrating clinical data, patient narratives, and technical components, an approach suitable for exploring complex healthcare interactions when traditional qualitative methods are unfeasible. Additionally, we analysed medical records from multiple sources, and the analysis of 1,116 healthcare contacts offered rich insights into documentation practices across settings and time periods. Qualitative thematic analysis was applied to patient-reported information to explore how patient perspectives were recorded or overlooked throughout the care pathway. Multiple researchers from clinical, sociological, and technical fields participated in data extraction, analysis, and validation, reducing individual bias and increasing reliability.

While this manual thematic analysis was strictly an analytical tool, this retrospective approach means the framework cannot be applied prospectively to live clinical workflows in its current form; yet it provides the foundation for real-time utility. Operationally, the framework is intended to be implemented by embedding the standardised ISO 13940 concepts as dedicated data entry fields within the EHR user interface. This allows clinicians to register patient-centred goals, values and preferences in a structured manner at the point of care.

### 4.5. Ethical considerations

All procedures were conducted in accordance with ethical guidelines and regulations, with approvals obtained from the relevant institutional review boards: The Regional Committees for Medical and Health Research Ethics (REC 2021/381577), The National Archives of Norway (Arkivverket 2023/ H-142), the Directorate for e-health (2023/H-142 – E1) and the Data Protection Officer at University Hospital of North Norway (2022/8209). Data from deceased patients were processed in compliance with national regulations regarding posthumous medical records. For living patients, written informed consent was obtained before accessing their records, and participants were informed that they could withdraw from the study at any time without consequences. To protect privacy, patient data were de-identified by a healthcare professional and stored securely with restricted access to maintain confidentiality. No re-identification was attempted.

Beyond formal compliance, equity and inclusion are critical considerations. In this study, much of the patient narrative was mediated through clinicians’ documentation rather than provided directly by the patients themselves. In addition, some patients’ voices can be less represented due to factors such as gender, culture, income, or health status [42].

## 5. Conclusion

Our study highlights significant gaps in the documentation and sharing of patient-centred information across different healthcare levels. Notably, our findings demonstrate strong support from patients and healthcare professionals for incorporating this information into care; however, systemic, organisational, and technical barriers persist. Overcoming these challenges requires a comprehensive approach involving policy reforms, organizational strategies, technological solutions, and enhanced collaboration at the patient-provider level. By integrating patient perspectives and fostering an environment that values this information in care decisions, health information systems can better support patient-centred, integrated care pathways. This could enhance care coordination, support shared decision-making, align healthcare delivery with patients’ needs and preferences, and ultimately improve patient outcomes.

## 6. Implications for Future Research

Future research should focus on incorporating patient perspectives into EHR systems and computerised care decisions, using larger sample sizes and patient interviews or surveys to validate recommendations. Testing the proposed VHR model in a real-world setting can assess its feasibility, usability, and effectiveness. Exploring artificial intelligence (AI) to predict care pathway steps by integrating patient-centred and clinical data could advance patient-centred care. Investigating how health and digital literacy influence patient-centred EHR systems may improve accessibility and effectiveness for patients with complex needs. Finally, evaluating the impact of patient perspectives on clinical outcomes and exploring incentive programmes to promote their adoption can provide valuable insights for policymakers and organisations, bridging gaps and advancing patient-centred, integrated care pathways.

## Data Availability

The data analysed in this study consists of highly sensitive historical and contemporary medical records and cannot be made openly available in a public repository due to strict privacy, legal, and ethical restrictions protecting patient confidentiality.
